# Supplementary material for: The Thermal Properties and Degradability of Chiral Polyester-Imides Based on Several l/d-Amino Acids
Source: Polymers (Basel). 2020 Sep 9;12(9):2053. doi: 10.3390/polym12092053 (PMC7570264; doi:10.3390/polym12092053)
Supplement: Supplementary file 1 [file polymers-12-02053-s001.pdf]

Supplementary Material

The Thermal Properties and Degradability of Chiral Polyester-Imides Based on Several L/D-Amino Acids

Chen Qi [1,2], Wenke Yang [1,2], Fuyan He [1,2] and Jinshui Yao [1,2,]*

[1] School of Materials Science & Engineering, Qilu University of Technology (Shandong Academy of Sciences), Jinan 250353, China; 1240835573@qq.com (C.Q.); wkyang@qlu.edu.cn (W.Y.); hefuyan555@163.com (F.H.)

[2] Shandong Provincial Key Laboratory of Processing & Testing Technology of Glass and Functional Ceramics, Jinan 250353, China

* Correspondence: yaojsh@qlu.edu.cn; Tel.: +86-531-89631226

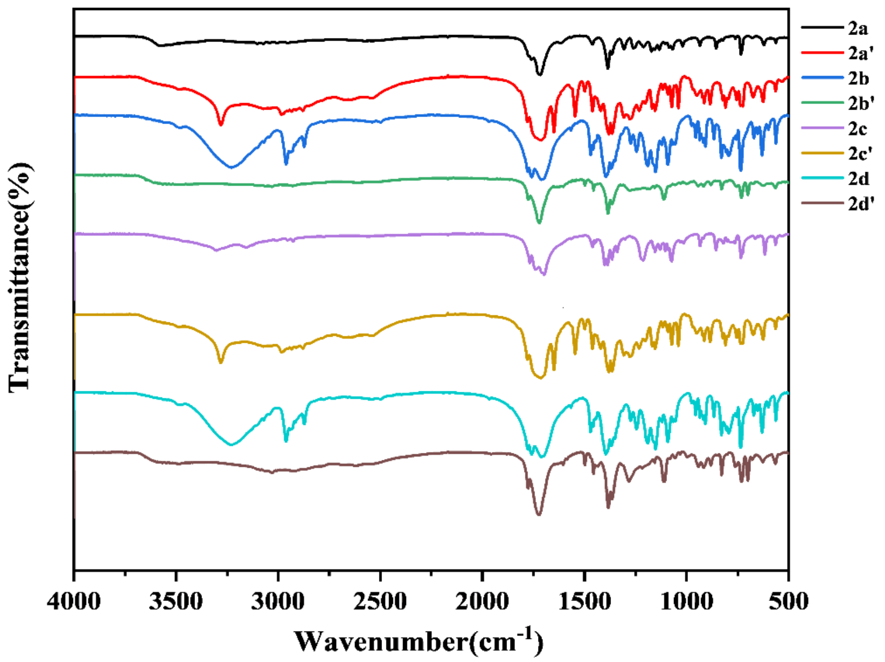

Figure S1. FTIR spectra of diacid monomers 2a-2d'.

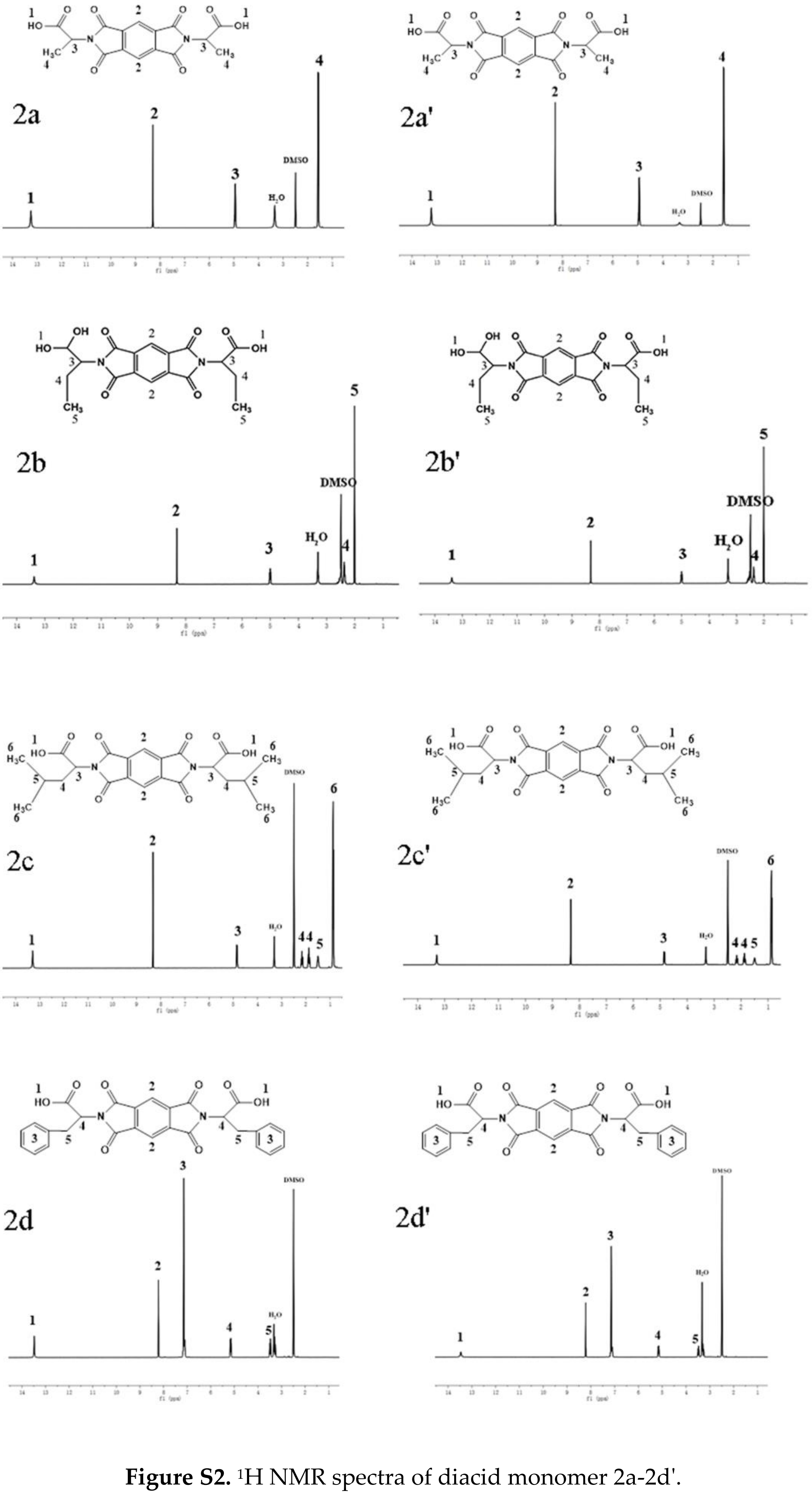

Figure S2. ^{1}H NMR spectra of diacid monomer 2a-2d'.

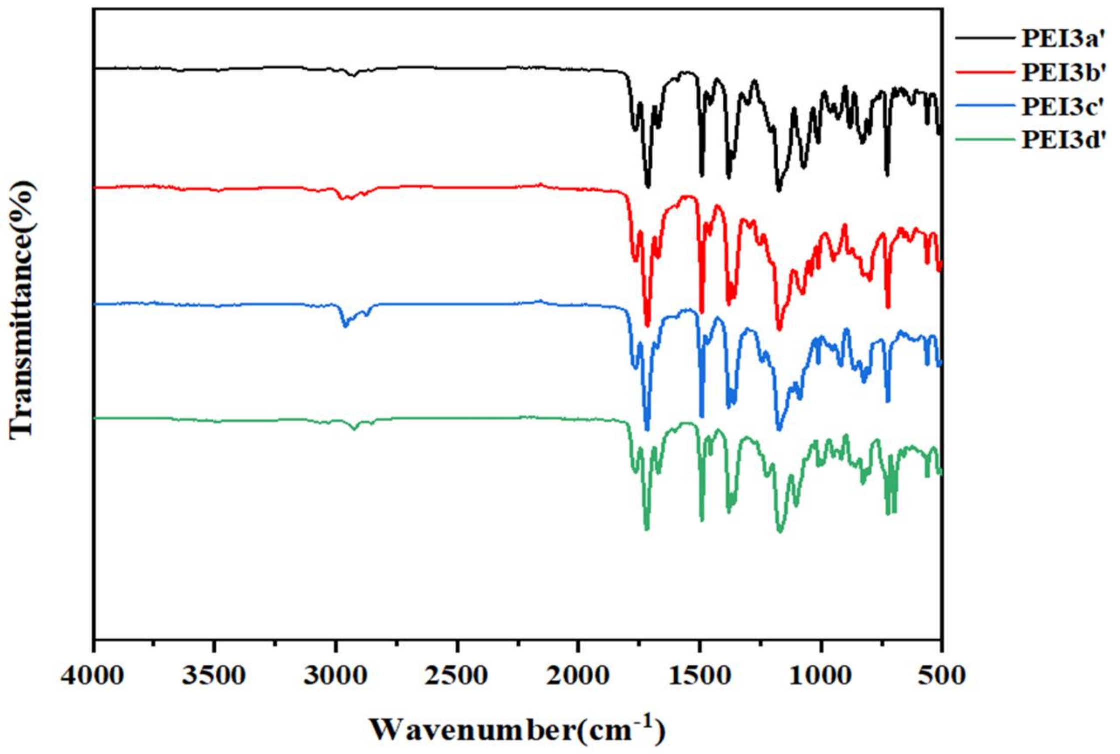

Figure S3. FTIR spectra of polyamide-imides PEI3a'-PEI3d'

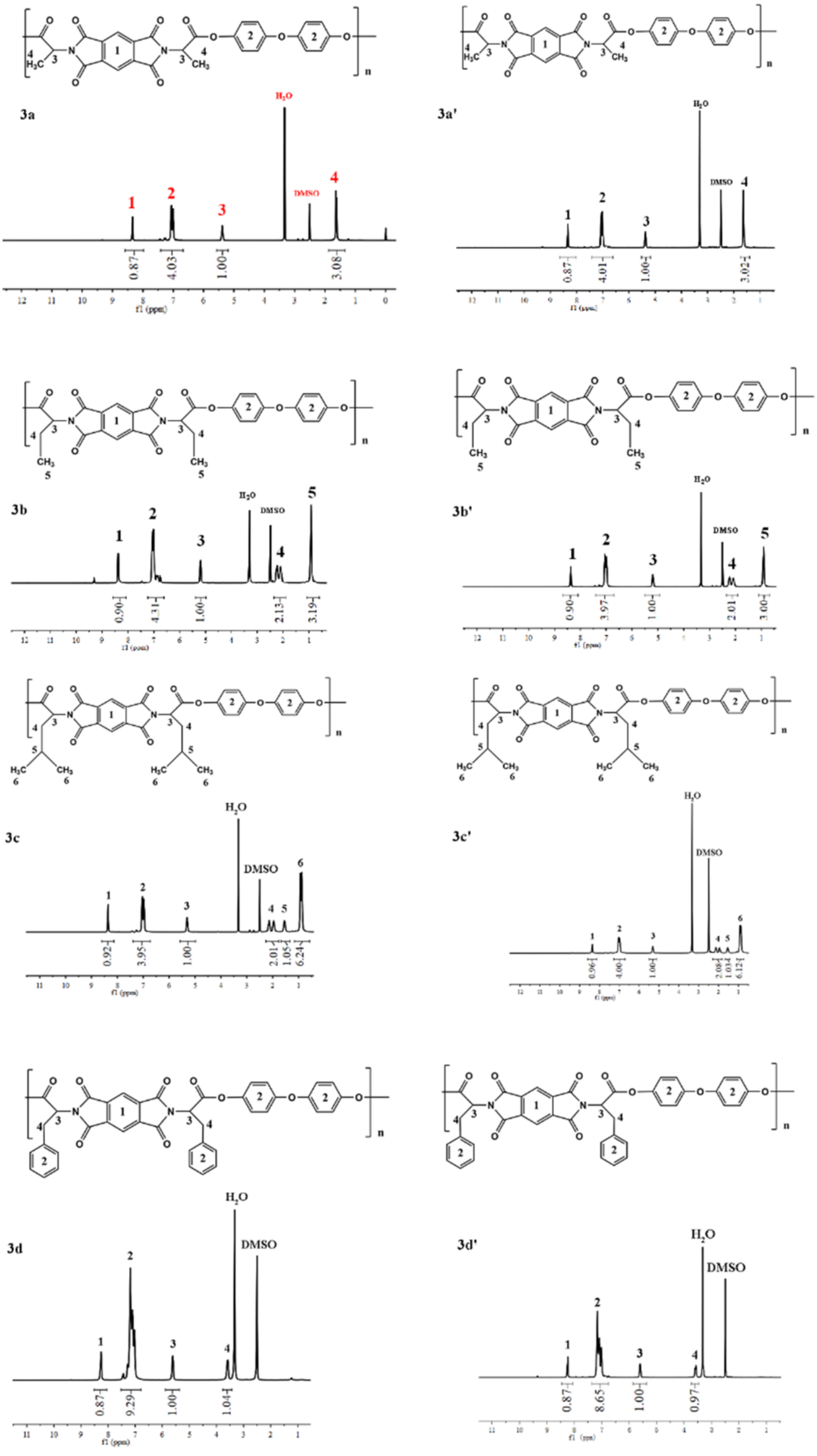

Figure S4. ^{1}H NMR spectra of polyamide-imides PEI3a-PEI3d'.
